# Ancestral Stem Cell Reprogramming Genes Active in Hemichordate Regeneration

**DOI:** 10.3389/fevo.2022.769433

**Published:** 2022-02-15

**Authors:** Tom Humphreys, Keith Weiser, Asuka Arimoto, Akane Sasaki, Gene Uenishi, Brent Fujimoto, Takeshi Kawashima, Kekoa Taparra, Janos Molnar, Noriyuki Satoh, Yusuke Marikawa, Kuni Tagawa

**Affiliations:** 1 Institute for Biogenesis Research, University of Hawai‘i at Mānoa, Honolulu, HI, United States; 2 Marine Biological Laboratory, Graduate School of Integrated Sciences for Life, Hiroshima University, Hiroshima, Japan; 3 Marine Genomics Unit, Okinawa Institute of Science and Technology Graduate University, Okinawa, Japan

**Keywords:** hemichordates, regeneration, stem-cell reprogramming genes, *Pou3*, *SoxB1*, *Msxlx*, *Klf1/2/4*

## Abstract

Hemichordate enteropneust worms regenerate extensively in a manner that resembles the regeneration for which planaria and hydra are well known. Although hemichordates are often classified as an extant phylogenetic group that may hold ancestral deuterostome body plans at the base of the deuterostome evolutionary line leading to chordates, mammals, and humans, extensive regeneration is not known in any of these more advanced groups. Here we investigated whether hemichordates deploy functional homologs of canonical Yamanaka stem cell reprogramming factors, *Oct4, Sox2, Nanog*, and *Klf4*, as they regenerate. These reprogramming factors are not expressed during regeneration of limbs, fins, eyes or other structures that represent the best examples of regeneration in chordates. We first examined *Ptychodera flava* EST libraries and identified *Pf-Pou3, Pf-SoxB1*, *Pf-Msxlx*, and *Pf-Klf1/2/4* as most closely related to the Yamanaka factors, respectively. *In situ* hybridization analyses revealed that all these homologs are expressed in a distinct manner during head regeneration. Furthermore, *Pf-Pou3* partially rescued the loss of endogenous Oct4 in mouse embryonic stem cells in maintaining the pluripotency gene expression program. Based on these results, we propose that hemichordates may have co-opted these reprogramming factors for their extensive regeneration or that chordates may have lost the ability to mobilize these factors in response to damage. The robustness of these pluripotency gene circuits in the inner cell mass and in formation of induced pluripotent stem cells from mammalian somatic cells shows that these programs are intact in humans and other mammals and that these circuits may respond to as yet unknown gene regulatory signals, mobilizing full regeneration in hemichordates.

## INTRODUCTION

The robust regeneration capacity of hemichordates, though known since the 19th century, has only recently received much attention as a model for studying regenerative processes (Humphreys et al., 2010; Miyamoto and Saito, 2010; Luttrell et al., 2016; Arimoto and Tagawa, 2018). Hemichordates, like planaria and hydra, can regenerate the whole body from relatively small pieces. A recent review suggests that very few other animal groups exhibit complete regeneration. Most are restricted to modest damage repair, such as severed appendages or eye damage (Agata and Inoue, 2012). On the other hand, mammalian reprogramming factors are not expressed in the modest examples of regeneration known in vertebrates, limb regeneration, for example. Although full regeneration in hemichordates can occur after a multitude of injuries, producing many different portions of their bodies (Humphreys et al., 2010; Miyamoto and Saito, 2010; Luttrell et al., 2016; Arimoto and Tagawa, 2018), we have focused on anterior regeneration after decapitation just posterior to the branchial basket to show that homologs of canonical reprogramming factors that produce mammalian induced pluripotent stem cells (iPSCs) (Takahashi and Yamanaka, 2006) function in regeneration of the acorn worm, *Ptychodera flava*.

**FIGURE 1 ∣:**
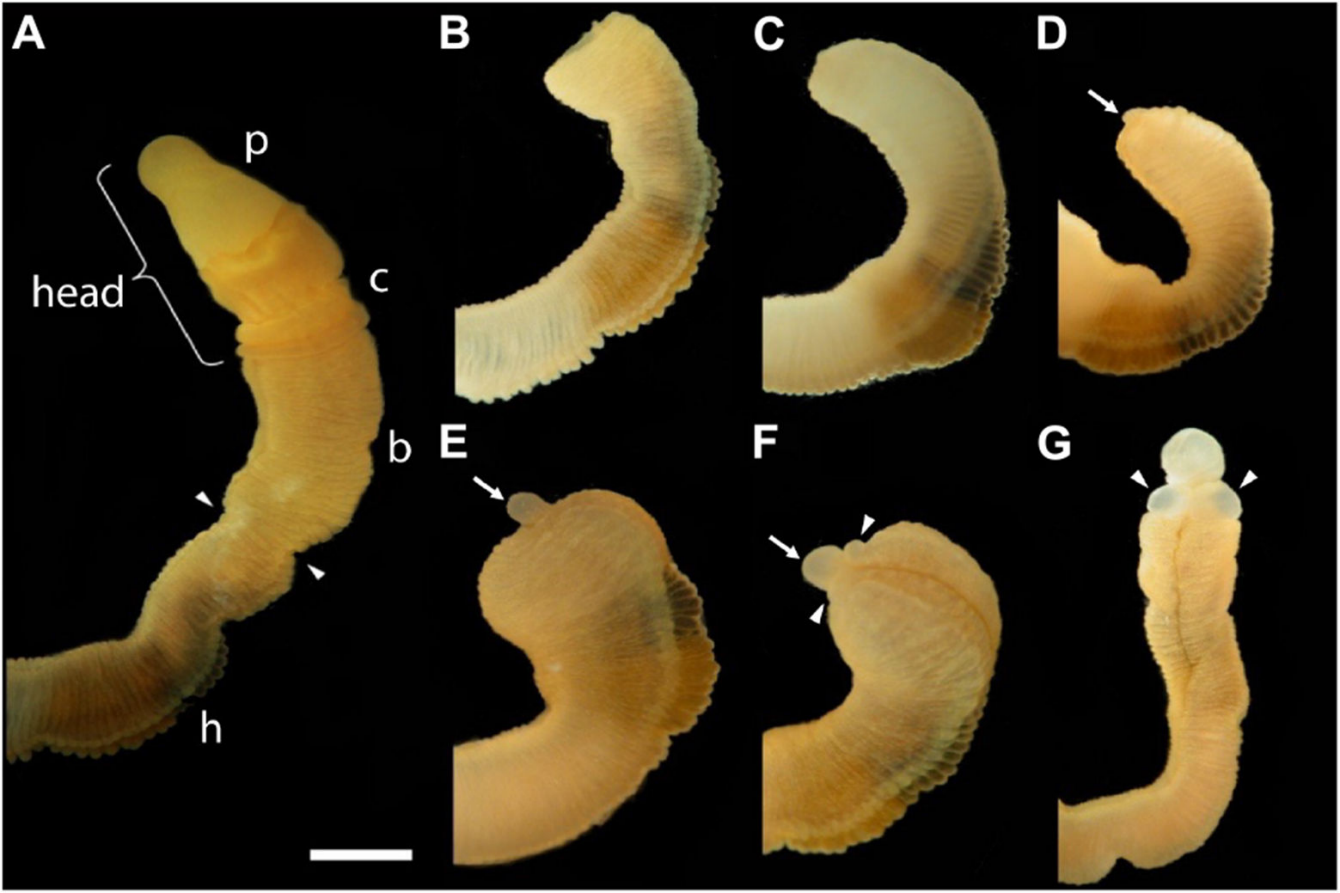
The time sequence of head regeneration of an individual *Ptychodera flava*. Note that *P. flava* worms are very hydraulic and the same animal may be extended and thin one moment and contracted and thick the next. **(A)** The uncut, intact animal. Arrowheads mark the site at posterior end of the gill basket where the animal was severed. Scale bar = 2 mm in all frames. **(B)** 0 days post-amputation (dpa); gaping anterior wound where the body wall has been severed. **(C)** 1 dpa; edges of the severed body wall are slightly swollen and smoothened and the wound has begun to close. **(D)** 3 dpa; wound has closed and a tiny blastema can be detected dorsal to the closure site (arrow). **(E)** 5 dpa; the blastema is growing rapidly (arrow). **(F)** 7 dpa; the blastema is shaped into a nascent proboscis (arrow) with a collar (arrowheads). **(G)** 11 dpa, the new head, approaching final size to match the original body, is attached to the original anterior cut stump. All views of the left side, except **(G)**, which is more ventral. Arrowheads show the newly regenerating collar. p, proboscis; c, collar; b, branchial region; h, hepatic region.

The first frame of Figure 1 shows an uncut animal with its original head, which includes the proboscis and collar, corresponding to the prosome and mesosome of the worm, respectively (Lacalli, 2005). Arrowheads mark the site where decapitation will occur (Figure 1A). The next six frames of Figure 1 show regeneration of this same animal over the next 11 days when an almost full-sized head has regrown on the cut stump. After the cut at 0 time, the body cavity and gut are a gaping wound to the outside environment (Figure 1B). By 2 days post-amputation (dpa), the edge of the wound becomes swollen, apparently from inflammation processes, and begins to pull closed (Figure 1C). At 3 dpa, the wound is closed and a cellular blastema has formed at the dorsal edge of the healed cut (shown with an arrow in Figure 1D). This blastema grows rapidly and by 5 dpa has the outline of a nascent head with a proboscis and collar surrounding a newly opened pharyngeal mouth (Figure 1E). The head continues to grow and differentiate on the stump of original tissue. Normal behavior of the animal is regained by day 9 or 10. By 11–14 dpa the new head, attached directly to the relatively unchanged stump of old tissue, approaches the diameter of the original body and stops growing (Figure 1G). Head regeneration is epimorphic on the stump of old tissue with little evidence of tissue reorganization.

In planaria, regeneration is based on neoblasts, a unique population of continuously dividing pluripotent stem cells that produce new cells in the planarian and form a regeneration blastema when part of the animal is removed (Morgan, 1900; Stephan-Dubois and Kolmayer, 1959; Elliott and Sánchez-Alvarado, 2013). In amphibian limb regeneration, a blastema forms, not from pluripotent stem cells, but apparently by dedifferentiation of limb tissue cells to multipotent stem cells (Kragl et al., 2009). Stem cells of various potency have been described in regeneration of other animals, such as crinoid echinoderms and colonial ascidians (Ferrario et al., 2020), but for most animals that show some regeneration, the role of stem cells is not well established. The role of stem cells or of dedifferentiation in hemichordate regeneration was unknown. Therefore, the present study examined the mode of regeneration with molecular probes.

## MATERIALS AND METHODS

### Animals and Regeneration Experiments

Specimens of *Ptychodera flava* were collected from areas of fine to coarse, clean sand on the extensive flat shallow reef at Paiko, Oahu, Hawaii, where tidal depths range from 0 to 0.7 m. Worms were exposed by disturbing the sand to a depth of 4–10 cm with a wave of the hand in the water over the sand. Worms ranged from 2 to 20 cm in length. About 2% of the worms collected exhibited signs of recent anterior regeneration irrespective of the season. Animals were maintained in the laboratory in 60-liter seawater aquaria filled with reconstituted sea water (Instant Ocean) at 26°C and 3 cm of coarse (1–3-mm grains) coral gravel on the bottom filter driven by aeration. The aquaria were kept clean, and 50% of the water was changed every 2 weeks.

Intact animals or anterior pieces with heads that had posterior parts removed by transection were kept in closed plastic boxes with tops and bottoms of heavy 1-mm mesh screen, covered with a layer of fine, clean sand (2–5 mm). Recently transected posterior bodies were kept in clean glass bowls in the aquaria until about 12 days of regeneration, at which point the animals regained burrowing behavior and could crawl out of the bowls. They were then moved to the closed plastic boxes with mesh tops and bottoms and a layer of fine sand. In all experiments, freshly collected animals were severed at various body levels with an orthogonal cut using sharp surgical scissors.

### 5-Bromo-2′-Deoxyuridine and 5-Ethynyl-2′-Deoxyuridine Labeling

5-bromo-2′-deoxyuridine (BrdU) was incorporated into developing blastemas by incubating *P. flava* at the specified regenerative day in individual 5-cm dishes containing 2 mL salinized water with 100 μM BrdU for 16 h at room temp. Severed blastema samples were fixed in 4% paraformaldehyde with 0.5 M NaCl, 0.1 M MOPS, pH 7.5 for 2 h on ice. Blastema samples were then washed sequentially in 30% EtOH, twice in 50% EtOH, and three times in 80% EtOH, for 5 min each at room temperature (RT), and stored in 80% EtOH at −80°C until they were used for paraffin embedding. On the day of embedding, samples were washed 3 × 10 min in 100% EtOH at RT, then 2 × 30 min in 100% xylene at RT, then once in 50% xylene 50% paraffin for 1 h at 60°C. Finally, samples were transferred to molds and washed 2 × 2 h in paraffin at 60°C, before allowing them to set in fresh paraffin, with the sample oriented for sectioning at 20 μm. Slides were rehydrated by washing sequentially 2 × 10 min in xylene at RT, 2 × 10 min in 100% EtOH at RT, in 75% EtOH for 5 min at RT, in 50% EtOH for 5 min at RT, and in 100% H_2_O for 5 min at RT. For antigen retrieval, samples were incubated in 10 mM citrate pH 6.0 for 35 min at 100°C. After cooling to RT for 20 min, samples were processed for immunohistochemistry (IHC). Samples were washed in PBS with 0.2% gelatin and 0.05% sodium azide and processed for IHC. The anti-BrdU antibody (OBT0030, Oxford Biotech) was used at a 1:200 dilution, while the secondary antibody was used at a 1:500 dilution. DAPI was used to visualize all nuclei.

*Ptychodera flava* were incubated with 100 μM 5-ethynyl-2′-deoxyuridine (EdU) in seawater to perform a pulse-chase reaction. EdU labeled cells were visualized following instructions supplied with the Click-iT EdU Alexa Fluor 594 Imaging Kit (Life Technologies, New York, NY, United States). To examine whether dividing cells moved or not, uninjured animals were pulsed for 24 h with 100 μM EdU in seawater. Animals were then decapitated and allowed to regenerate for 48 h. Moreover, animals were pulsed for 6 h at 3 days after decapitation and chased for 24 h.

Samples were fixed in 4% paraformaldehyde overnight and dehydrated in EtOH. Then, samples were rehydrated and embedded in 4% agarose and sectioned at 50 μm. Sections were blocked with 3% BSA for 30 min, permeabilized with 0.5% Triton X-100, and then washed twice with 3% BSA for 30 min. Finally, sections were incubated with Click-iT reaction buffer (CuSO_4_, Alexa Fluor 594 azide, ascorbic acid) for 2 h.

### *In situ* Hybridization

Fixation of samples and *in situ* hybridization were carried out as previously described for hemichordate embryos and larvae (Tagawa et al., 1998; Lowe et al., 2004) using probes for *Pf-SoxB1* (Taguchi et al., 2002), *Pf-Pou3, Pf-Msxlx, Pf-Klf1/2/4, Pf-Gsc* (our present study), and *Pf-FoxA* (Taguchi et al., 2000). Orthology of *Pf-Pou3, Pf-Msxlx* and *Pf-klf1/2/4*, newly isolated genes in this study, was determined using neighbor joining and maximum likelihood methods (details in the Supplementary Material). The sequence of *Pf-Gsc* was identical to that previously reported by Su et al. (2019). Nucleotide sequence data reported here are available in the DDBJ/EMBL/GenBank database under the following accession numbers: LC622252 for *Pf-Pou3*, LC622253 for *Pf-Msxlx*, LC622254 for *Pf-Klf1/2/4*, LC622255 for *Pf-Gsc*, AB894822 for *Pf-SoxB1*, and AB023019 for *Pf-FoxA*. Sources of sequences are also available in the Supplementary Material.

### Functional Assay of *Pf-Pou3*

A mouse ES cell line ZHBTc4 (Niwa et al., 2000), a gift from Dr. Hitoshi Niwa (RIKEN Center for Developmental Biology, Kobe, Japan) was cultured in ESGRO Complete Clonal Grade Medium (Millipore, Billerica, MA, United States) without feeder cells on gelatin-coated dishes. In the ZHBTc4 cell line, the *Oct4* gene promoter was engineered to respond to TetR (tetracycline-dependent transcriptional repressor), so that endogenous *Oct4* expression could be suppressed by addition of tetracycline to the culture medium (Niwa et al., 2000). cDNA encoding *Pf-Pou3* was inserted in the multiple cloning site of the pCAG-IP vector (a gift from Dr. Niwa), which contains the CAG promoter, the internal ribosome entry site, and the puromycin resistance gene. The CAG promoter consists of the cytomegalovirus early enhancer element and the chicken beta-actin gene promoter, and its activity is not affected by tetracycline. For transfection, 5 × 10^4^ cells were seeded in each well of 24-well plates, and plasmids (800 ng per well) were transfected the following day using Lipofectamine 2000 (Invitrogen, Carlsbad, CA, United States) according to manufacturer instructions, followed by antibiotic selection (puromycin) for over 2 weeks to obtain stably transfected cell lines. Endogenous *Oct4* expression in ZHBTc4 cells was suppressed by addition of tetracycline at 10 ng/mL for 24 h. Cells were then harvested for gene expression analyses by quantitative RT-PCR. Total RNA was extracted using TRI reagent (Invitrogen) and used for cDNA synthesis with oligo dT primer and M-MLV Reverse Transcriptase (Promega). Real-time PCR was performed using iCycler Thermal Cycler with MyiQ Single Color Real-Time PCR Detection System (Bio-Rad, Hercules, CA, United States) with iQ SYBR Green Supermix (Bio-Rad). *GAPDH* levels were used to normalize expression levels of *Oct4*-dependent genes, namely *Rex1, Fgf4*, and *Klf4*. Experiments were repeated four times, and data are presented as means ± SD.

## RESULTS

### Where Do Blastema Cells Originate?

**FIGURE 2 ∣:**
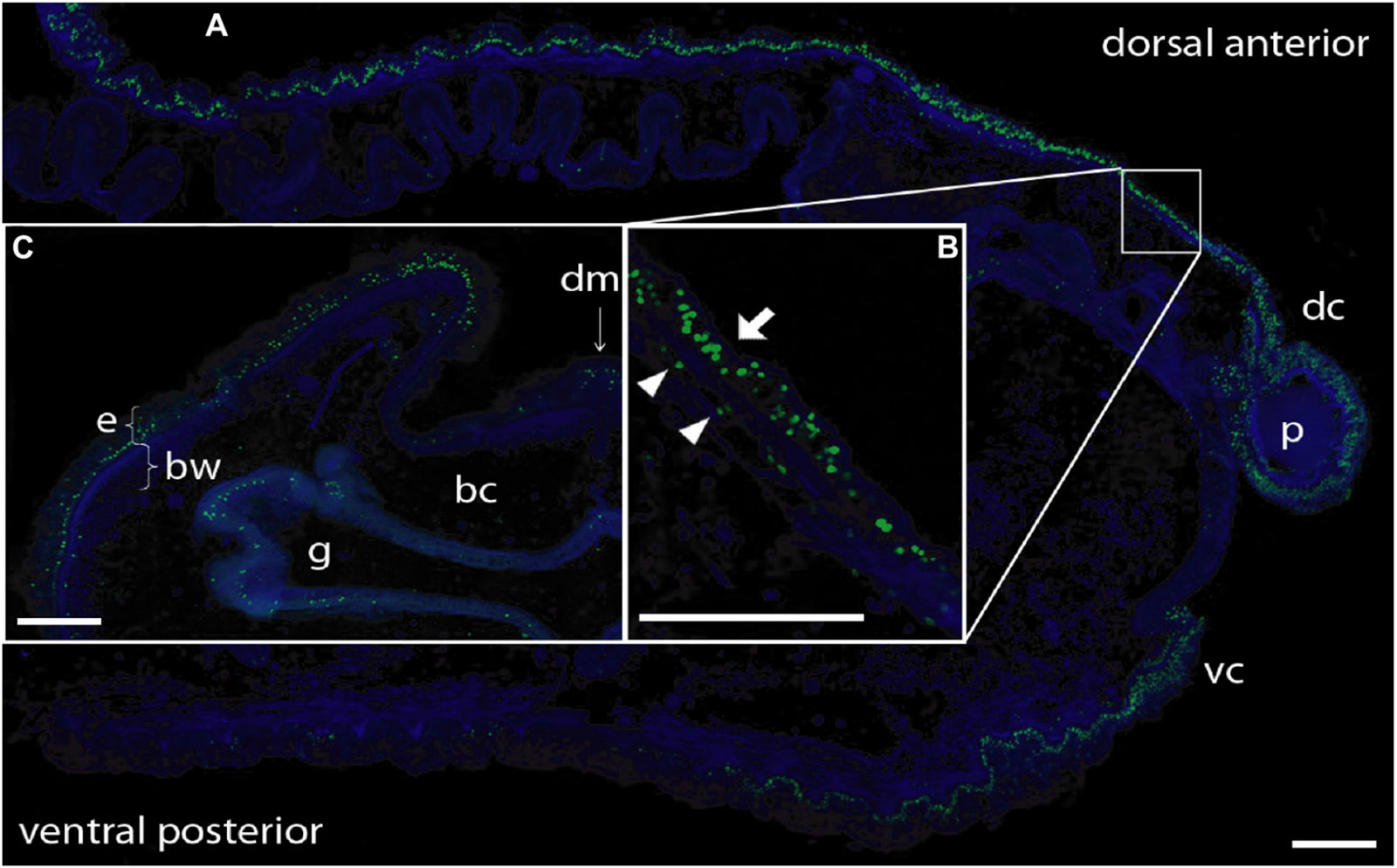
BrdU labeling of nuclei during anterior regeneration. Green indicates BrdU-labeled nuclei. Blue is DAPI staining for all nuclei. Scale bars = 0.3 mm. **(A)** Anterior sagittal section of an animal with a regenerating proboscis at 6 days post-amputation (dpa). Many nuclei synthesizing DNA are distributed in the blastema/regenerating proboscis and in cells of the ectodermal columnar epithelium around the blastema, extending considerably more posterior in the dorsal epithelium. **(B)** Inset: Enlargement of the area from the small square in **(A)**. Most labeled nuclei are in the epithelium (arrow), but there is a small set of mesenchymal-labeled nuclei (arrowheads) below the basement membrane of the epithelium (staining of basement membrane not shown in this image). **(C)** Lateral dorsal quadrant of a body cross section posterior to a regenerating blastema at 5 dpa. Nuclei incorporating BrdU are distributed in the dorsal lateral epithelium from the dorsal midline to the lateral edge. The ventral epithelium (not shown) contains few labeled nuclei. Labeling in the gut wall is the same as in non-regenerating animals. Acquired microscopic data were composed into a single image for each sample. bc, body cavity; bw, body wall (mesodermal); dc, dorsal collar; dm, dorsal mesentery; e, epithelium (ectodermal); g, gut cavity; p, proboscis; vc, ventral collar.

To determine the provenance of dividing cells in hemichordate regeneration, we first employed BrdU labeling at various times of regeneration. By 48 h, cell division is apparent in the dorsal epidermis proximal to the decapitation cut and by 72 h the population of dividing cells is even more prominent in the blastema that has formed and in the dorsal epidermis proximal to the original cut (data not shown). As shown in Figure 2, at 6 dpa of regeneration, there is a prominent population of dividing cells in the dorsal epidermis, about 3–4 mm proximal to the regenerating proboscis and in the regenerating portion itself. At this point, most dividing cells in the original tissue appear to be in epidermal ectoderm (see arrow in Figure 2B), but some are below the basement membrane among the mesodermal cells (see arrowheads in Figure 2B).

**FIGURE 3 ∣:**
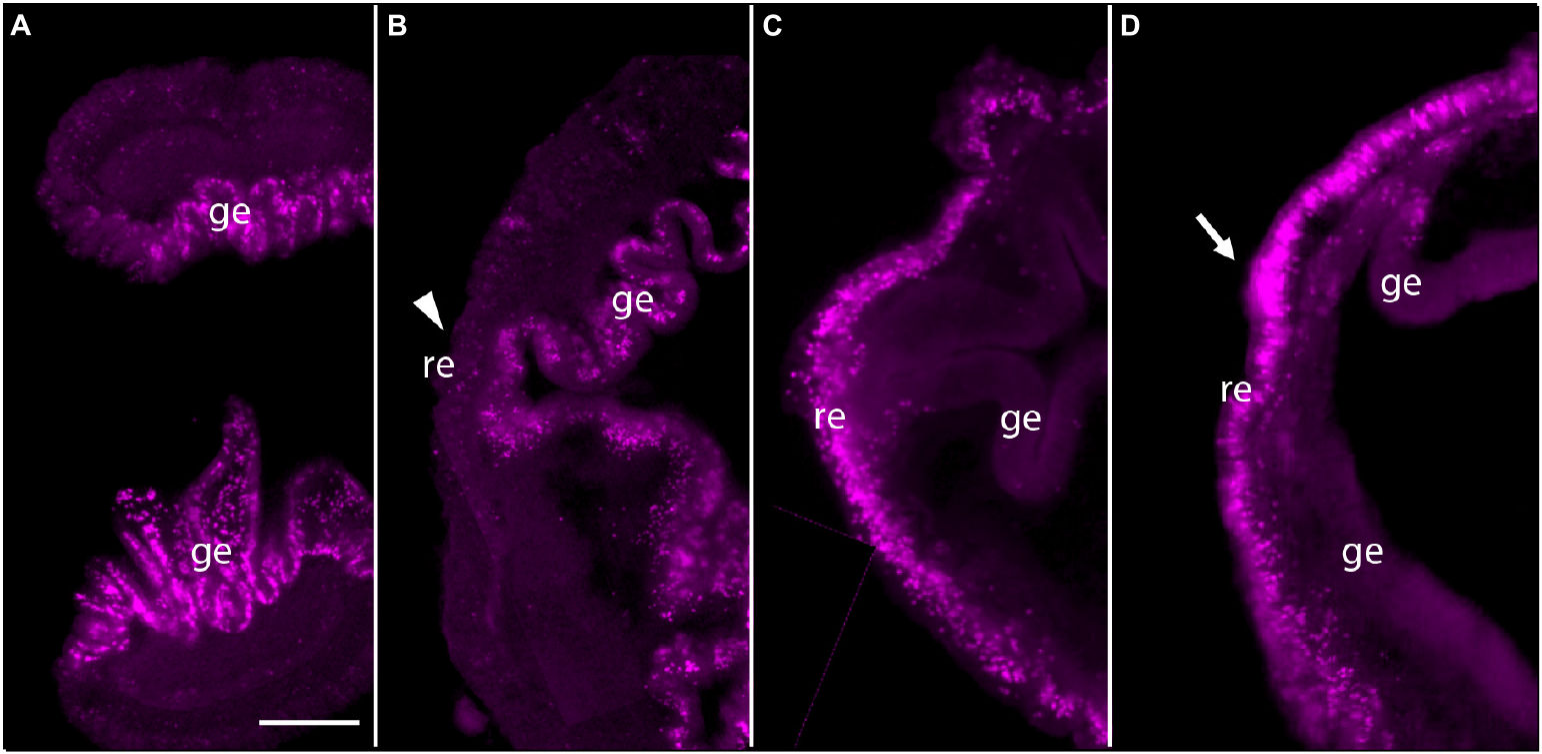
Pulse-Chace experiments using EdU. **(A)** Uninjured animals were incubated with EdU and dividing cells were detected. Most dividing cells were observed in the gut epithelium. **(B)** Uninjured animals were incubated with EdU, then cut and allowed to regenerate for 48 h. There were very few labeled cells in anterior regenerating tissue (arrowhead), but many labeled cells in the gut epithelium. **(C)** Animals were labeled with EdU at 3 days post-amputation (dpa) and dividing cells were detected. Labeled cells were detected in and around the epithelium of regenerating tissues. **(D)** Animals were labeled with EdU at 3 dpa and allowed to regenerate for 24 h. Labeled cells appeared to remain in the anterior portion of the regenerating tissue after 24 h. The mass of labeled cells enlarged, and the regenerating blastema appeared to start forming a small proboscis (arrow). The left side is the anterior portion of regenerating tissues in all panels. Scale bar = 0.3 mm and all samples are 3–4 mm in size. Acquired microscopic data were composed into a single image for each sample. ge, gut epithelium; re, regenerating epithelium.

Then we employed EdU labeling at earlier stages of regeneration. When intact animals are labeled with EdU, virtually all labeled cells appear in the gut epithelium (Figure 3A). If EdU was pulsed and removed from intact animals and then the animals were decapitated and allowed to regenerate, these previously labeled cells remain mostly in the gut and do not appear to contribute to subsequent blastema formation (Figure 3B). In contrast, if EdU was pulsed and removed from the animal at 3 dpa, labeled cells were mainly detected in regenerating epithelium and its vicinity, not in the gut epithelium (Figure 3C). After 24 h, labeled cells appeared to remain in the anterior portion of regenerating tissues and the mass of labeled cells enlarged especially in the nascent proboscis (Figure 3D; arrow), although labeled cells increased in gut epithelium compared to the earlier stage of regeneration (Figures 3C,D). These observations of dividing cells during anterior regeneration suggest that cells contributing to hemichordate regeneration come predominantly from dorsal ectodermal epithelium and ventral ectodermal epithelium in the vicinity of the original amputation.

### Do Blastema Cells Express Reprogramming Factors?

#### Hemichordate Homologs of Mammalian Reprogramming Factors

A full set of functional reprogramming factor genes, *Sox2, Klf4, Oct4*, and *Nanog*, has not been identified in any group except mammals, even though orthologs of *Oct4* and *Nanog* have been identified in the ancestral line of vertebrates (Theunissen et al., 2011; Onichtchouk, 2012). Although hints of the basics of a pluripotent gene program have been suggested in planaria and hydra, it seems that an *Oct4*-centric program is not present in these animals (Eisenhoffer et al., 2008). As the crucial lineage in the evolutionary line leading to mammals, it may be possible that hemichordates carry comparable gene programs. To identify and characterize hemichordate homologs of the four mammalian reprogramming factor genes, we carried out BLAST searches of *P. flava* EST libraries with approximately 160,000 independent sequences (Tagawa et al., 2014). When we found candidate genes, we confirmed them by molecular phylogenetic analysis (Takahashi et al., 2007; see Supplementary Material). First, together with results of previous studies (Taguchi et al., 2002; Zhong et al., 2011), the orthology of *Pf-SoxB1* with mammalian *Sox2* is evident. Second, molecular phylogenetic analysis demonstrated the orthology of *Pf-Klf1/2/4* with mouse and human *Klf4* (Supplementary Figures 1, 2).

On the other hand, the presence of *Oct4* and *Nanog* orthologs in invertebrates requires careful characterization, since no studies have identified *Oct4* nor *Nanog* sequences except in vertebrates, not even in invertebrate chordates. As to *Oct4*, an ancient trait of Oct4 and Pou proteins has been suggested (Holland et al., 2007; Tapia et al., 2012). Of the six classes of Pou transcription factors, classes I, III, IV, and VI were already present in the last common ancestor of metazoan and the PouII class was recovered only from bilaterians and the PouV class only from vertebrates (Gold et al., 2014). Both PouII and PouV class genes are most likely evolved from PouIII (Onichtchouk, 2016). We found that the *P. flava* genome contains members of classes II, III, IV, and VI. We named a hemichordate gene in the Pou3 subclass, *Pf-Pou3* (Supplementary Figures 3, 4). Mammalian *Oct4* (*Pou5f1*) has a sequence that is most closely related to the human and mouse *Pou3* genes, since Pou3 and Pou5 formed a clade in the molecular phylogenetic tree (Supplementary Figures 3, 4). That is, the characterization of *Pou* homeobox genes among all human homeobox genes suggests that *Pou5* (*Pou5f1* or *Oct4*) and *Pou3* evolved from a shared ancestral gene (Holland et al., 2007; Tapia et al., 2012). Therefore, we conclude that *Pf-Pou3* is a homolog of mammalian *Oct4*. This homology was supported by a functional assay of *Pf-Pou3*, as will be described later (see section “Functional Assay of Pf-Pou3 in vitro”).

The aforementioned sequences orthologous to *Nanog* have not been found outside of vertebrates. Our previous study demonstrated that highly significant matches of *Nanog* were found only in homeodomains. The seven best matches encoded genes containing NK-subclass homeodomain sequences (Molnar et al., unpublished data). Further phylogenetic analysis indicated that these sequences represent *P. flava* orthologs of the NKL-subclass genes, *Msxlx, Msx, Dlx, NK2.1, NK2.3, NK5*, and *Tlx*. Of them, *P. flava Msxlx* was shown in phylogenetic trees to be most closely related to human *Nanog*. Since *Nanog* is not present in invertebrates, we carried out molecular phylogeny of invertebrate *Msxlx* genes and found that *Pf-Msxlx* is a hemichordate member of *Msxlx* genes (see Supplementary Figures 5, 6). In summary, *Pf-SoxB1, Pf-Klf1/2/4, Pf-Pou3*, and *Pf-Msxlx* comprise a homologous set of mammalian reprogramming factor genes, *Sox2, Klf4, Oct4*, and *Nanog*.

#### Expression of *Pf-Pou3, Pf-Msxlx, Pf-SoxB1, and Pf-Klf1/2/4* During Regeneration

**FIGURE 4 ∣:**
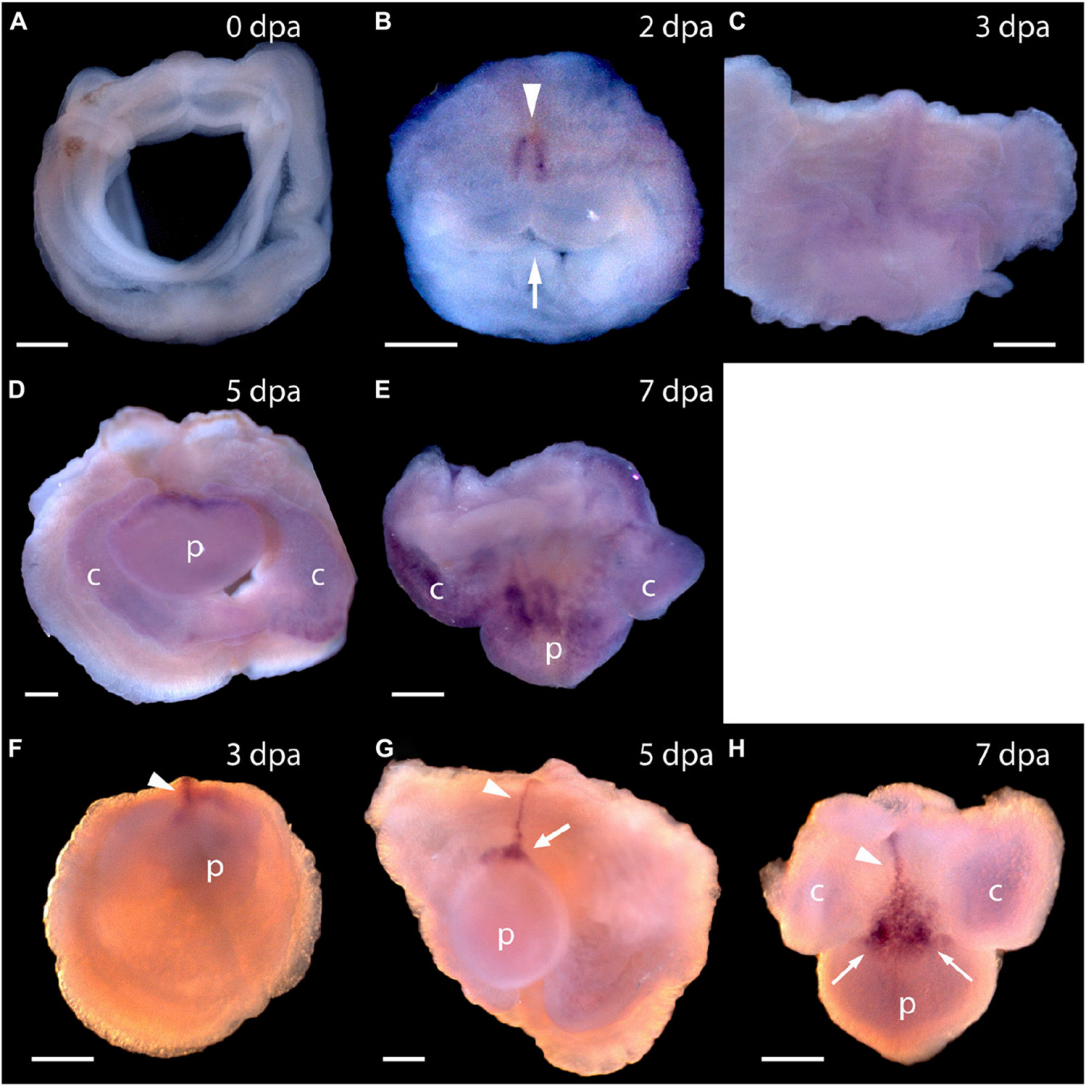
Expression of *Pf-Pou3* and *Pf-Msxlx* during head regeneration. **(A–E)** Whole mount **in situ** hybridization of *Pf-Pou3*. **(A)** At 0 days post-amputation (dpa), no signal is detected in the tissue at the site of transection. **(B)** At 2 dpa, signal is first detected along the dorsal mid-line (arrowhead) just posterior to the site of transection. Note that at 2 dpa the wound edges are swollen and the wound is still open to the gut (arrow). **(C)** At 3 dpa, the wound has closed and signal along the dorsal midline has extended anteriorly into the base of the nascent blastema. **(D)** At 5 dpa and **(E)** at 7 dpa (dorsal view), a strong signal at the base of the blastema and forming head continues during the course of regeneration. At this time, a weak signal appears throughout the blastema and forming head. **(F–H)** *In situ* hybridization of *Pf-Msxlx*. **(F)** At 3 dpa, signal is evident along the dorsal nerve track (arrowhead). **(G)** At 5 dpa and **(H)** at 7 dpa (dorsal view), signal has extended along the dorsal mid-line to the base of the blastema where it widens into a triangle (arrows). This pattern of expression continues as the new head develops. p, nascent proboscis; c, nascent collar. Dorsal is up in all frames, unless noted. Scale bars = 0.25 mm.

**FIGURE 5 ∣:**
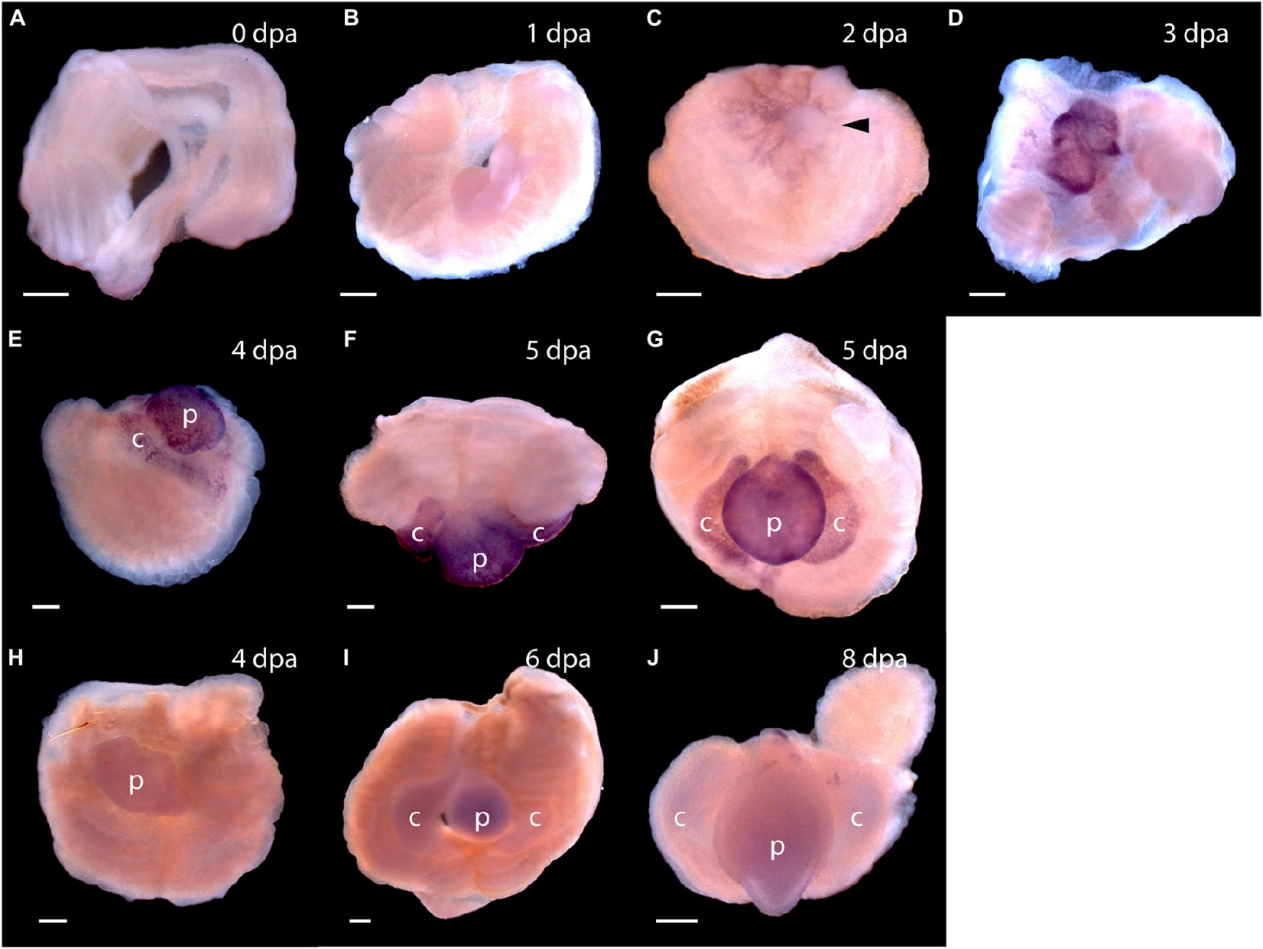
Expression of stem cell genes *Pf-SoxB1* and *Pf-Klf1/2/4* during head regeneration. **(A–G)** Whole mount *in situ* hybridization of *Pf-SoxB1*. At 0 days post-amputation (dpa) **(A)** and at 1 dpa **(B)**, no signal is detected. **(C)** At 2 dpa, a fine, reticulated signal appears at the swollen edges of the cut body wall being pulled into the closing wound. The arrowhead marks an unlabeled, rounded bleb of swollen body wall not yet pulled into the wound. **(D)** At 3 dpa, a strong signal appears only in the blastema. This blastema appears as two parts. These parts usually merge into one as they grow. **(E)** At 4 dpa, signal appears only in blastema with signal showing in the nascent collar as well as in the proboscis. **(F)** At 5 dpa, dorsal view, signal appears only in the blastema with no signal along the dorsal midline where *Pf-Pou3* and *Pf-Msxlx* signals are prominent (see Figures 4A-G for *Pf-Pou3* and Figures 4F-H for *Pf-Msxlx*). **(G)** At 5 dpa, there is signal throughout the blastema, but no signal in the dorsal trunk. Dorsal is up in all frames except **(F)** where the dorsal surface is facing the viewer. **(H–J)** *In situ* hybridization of *Pf-Klf1/2/4*. **(H)** At 4 dpa, **(I)** at 6 dpa, and **(J)** at 8 dpa, definite, but weak signal spread generally throughout the blastema and regenerating tissue. p, nascent proboscis; c, nascent collar. Scale bars = 0.25 mm.

To answer the question of whether these genes are expressed in acorn worm regeneration, we made antisense probes for whole mount *in situ* hybridization from each of the *P. flava* sequences and reacted these probes with fixed tissue from regenerating individuals at various days following body transection, using *in situ* protocols developed for embryonic tissue (Tagawa et al., 1998; Humphreys et al., 2010). Sense probes were made for control reactions in all cases and as expected, did not produce a signal. *In situ* hybridization of tissue at the site of transection, 0 or 24 h after surgery, produced no signal in the cut stump from any of these probes or at 0 dpa for *Pf-Pou3* (Figure 4A) or at 0 dpa and 1 dpa for *Pf-SoxB1* (Figures 5A,B). From 2 - 4 dpa, while wound closure is occurring and a blastema is established, all probes began to produce signals, but in two distinct patterns. Before a signal is evident in the blastema, signals from *Pf-Pou3* and *Pf-Msxlx* appear in the original tissue along the dorsal midline, just posterior to the site of the original transection (Figures 4B,C). As described above, the dorsal epidermis is the location of early dividing cells when regeneration begins (Figures 2, 3). As regeneration proceeds, initial expression of *Pf-Pou3* in the medial dorsal tissue of the old body wall posterior to the cut extends anteriorly and becomes prominent at the dorsal base of the blastema (Figures 4C,D). This expression in the dorsal base of the blastema continues during growth and differentiation of the blastema and head (Figure 4E). Although the most prominent expression of *Pf-Pou3* occurs in the dorsal surface of the original body wall and in the base of the blastema, there may be slight signal throughout the blastema and regenerating head during the course of regeneration (Figures 4D,E). The initial dorsal midline signal from the *Pf-Msxlx* probe also becomes more prominent, extends up to the dorsal base of the blastema, and spreads a bit laterally at the base of the blastema (Figures 4F-H), but only a small signal-positive region is evident in the dorsal base of the blastema and does not extend into the regenerating head (Figures 4G,H).

As soon as the blastema is evident, signals from *Pf-SoxB1* and *Pf-Klf1/2/4* show as general signals throughout the blastema (Figure 5D for *Pf-SoxB1* and Figure 5H for *Pf-Klf1/2/4*). Some *Pf-SoxB1* signal may appear at 2 dpa in a reticulated pattern around the swollen edges of the cut body wall (Figure 5C) contracting together to close the wound. As soon as the wound opening has sealed and a blastema is evident, *Pf-SoxB1* gives a strong signal distributed throughout the blastema, and only in the blastema (Figures 5D-G). Although the *Pf-SoxB1* signal is very bright in the blastema, *Pf-SoxB1* produces no evident signal along the dorsal midline where *Pf-Pou3* and *Pf-Msxlx* signals are prominent (see Figures 4B-E for *Pf-Pou3* and Figures 4F-H for *Pf-Msxlx*, which are oriented to prominently display the dorsal midline of the preparation). As with *Pf-SoxB1*, the *Pf-Klf1/2/4* signal is distributed throughout the blastema and regenerating tissue at all times from the time when the blastema can first be recognized until head formation approaches completion (Figures 5H-J). In our hands, the *Pf-SoxB1* signal is always much brighter than the *Pf-Klf1/2/4* signal (compare Figures 5A-G with Figures 5H-J).

### Functional Assay of *Pf-Pou3 in vitro*

Examination of the ancestral origin of *Oct4* and its role in mammalian pluripotency has shown that homologs of the *Oct4* gene occur among all classes of vertebrates. These genes from lower vertebrates function in mouse embryonic stem (ES) cells to replace the function of the mouse *Oct4* gene when the latter is silenced (Tapia et al., 2012). We took the same approach, asking if *Pf-Pou3* could replace the function of mouse *Oct4* gene in mouse ES cells. We used mouse ZHBTc4 stem cells in which the endogenous *Oct4* gene was deleted, but that contain a mouse *Oct4* transgene that can be suppressed with tetracycline (Niwa et al., 2000). Cells were transfected with a hemagglutinin (HA)-tagged *Pf-Pou3* gene under control of a CAG promoter.

**FIGURE 6 ∣:**
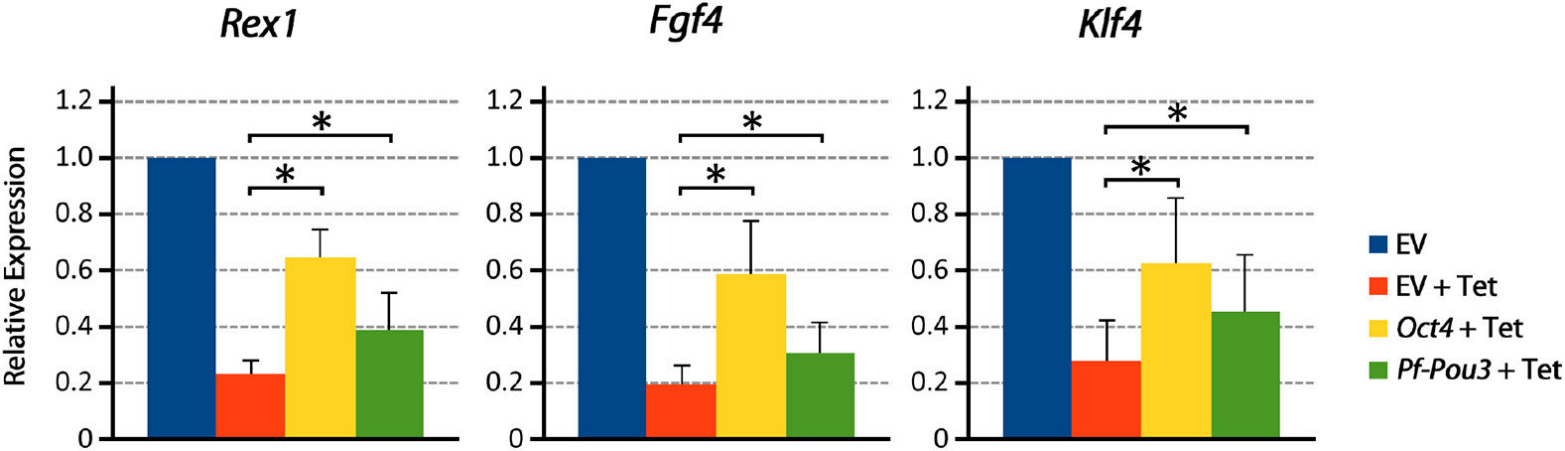
*Pf-Pou3* maintains expression of stem cell genes on loss of *Oct4* in mouse embryonic stem cells. ZHBTc4 cells were stably transfected with vectors constitutively expressing mouse *Oct4, P. flava Pou3 (Pf-Pou3)*, or empty vector negative control (EV). Stable cell lines were then grown for 24 h in the presence or absence of tetracycline (Tet). The graph shows the results of qPCR on cDNA generated from these cells measuring *Oct 4*-associated stem cell genes *Rex1* (*Zfp42*), *Fgf4*, and *Klf4*, displayed as quantities relative to negative control (EV). Asterisks represents statistical significance determined with the Mann–Whitney *U* test (*p* < 0.05). Expression of target genes was significantly reduced in the absence of *Oct4* and *Pf-Pou3*, but expression of all *Oct4*-associated genes tested was significantly recovered by stable transfection of mouse *Oct4* or hemichordate *Pf-Pou3*.

Immunohistochemistry with HA antibody showed that *Pf-Pou3* protein is expressed in these cells and is localized to the nucleus (data not shown). As shown in Figure 6, *Pf-Pou3* supports short-term expression of several *Oct4*-dependent genes that otherwise are not expressed when the endogenous mouse *Oct4* is turned off with tetracycline. Expression of three *Oct4*-dependent genes, *Rex1, Fgf4*, and *Klf4* as measured by qPCR, was decreased considerably when *Oct4* expression was turned off, but was maintained for 24 h upon expression of *Pf-Pou3*. This result indicates a functional conservation between *Pf-Pou3* and *Oct4*, supporting homology of the hemichordate and vertebrate genes.

### Are Hemichordate Blastema Cells Pluripotent?

**FIGURE 7 ∣:**
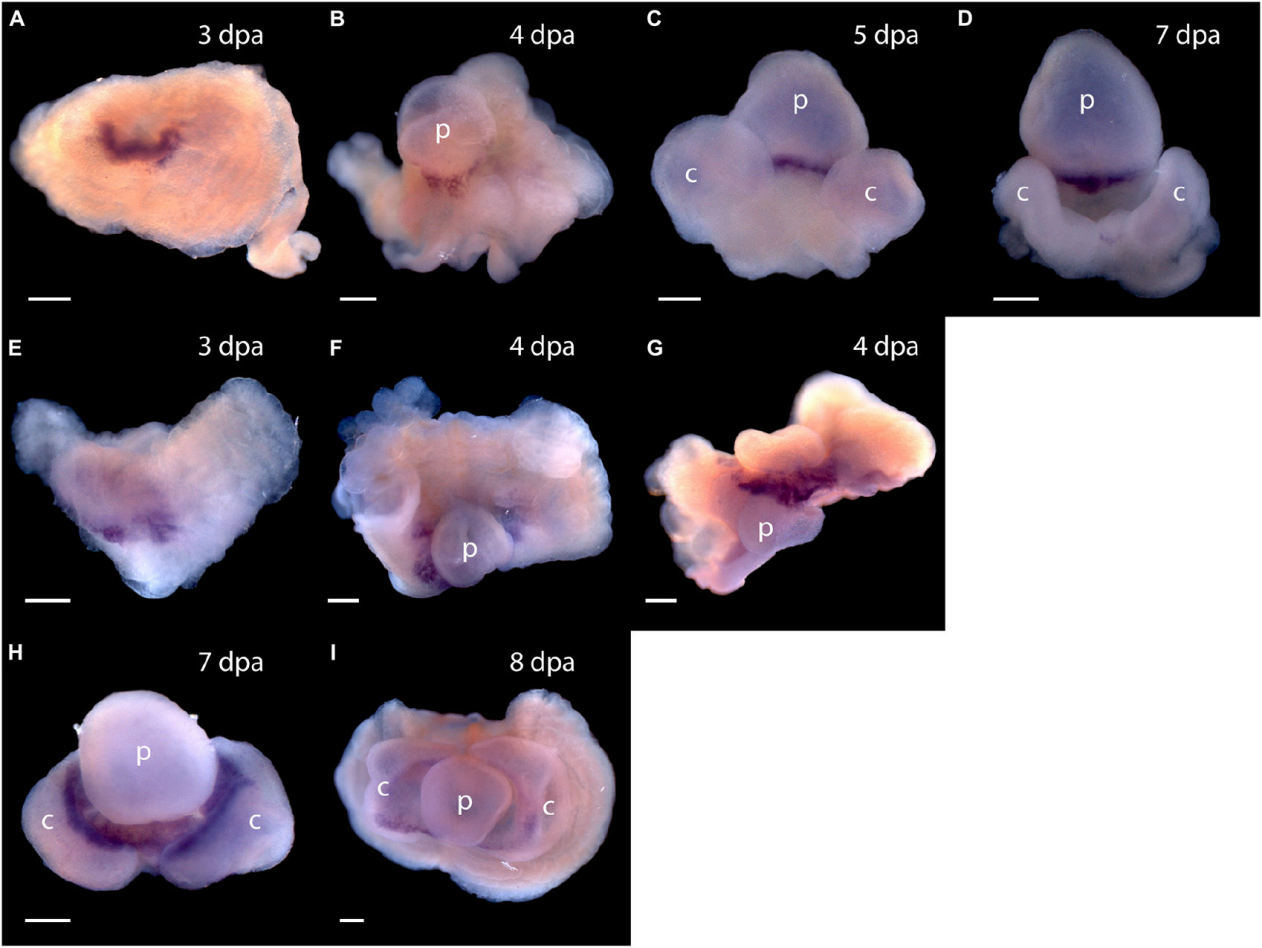
Expression of organizer genes *Pf-Gsc* and *Pf-FoxA*. **(A–D)** Whole mount *in situ* hybridization of *Pf-Gsc* at **(A)** 3 days post-amputation (dpa), **(B)** 4 dpa, **(C)** 5 dpa, and **(D)** 7 dpa of regeneration, respectively. *Pf-Gsc* marks the ventral base of the blastema, which become the base of the proboscis in all samples stained. **(E–I)** Whole mount *in situ* hybridization of *Pf-FoxA* at 3–8 dpa of regeneration. **(E)** *Pf-FoxA* is expressed at the ventral margin of the blastema, lateral to the *Pf-Gsc* signal, which becomes the edge of collar at 3 dpa. **(F)** At 4 dpa, dorsal view **(G)** at 4 dpa, **(H)** at 7 dpa, **(I)** at 8 dpa, ventral view. Ventral is facing the viewer in all figures except **(F,G)** in which the proboscis is facing the viewer. p, nascent proboscis; c, nascent collar. Dorsal is up in all panels except panel **(G)**. Scale bars = 0.25 mm.

Expression of *Pou3, Msxlx, SoxB*, and *Klf1/2/4*, homologs of mammalian reprogramming factors, in the regeneration blastema of *P. flava* suggests these cells may be pluripotent. How might we begin to test this idea? We thought it is possible that as cells of the *P. flava* head blastema initiate differentiation, they express sets of genes similar to what pluripotent mouse epiblast cells express as they initiate development. Two of the first genes expressed in the inner cell mass as the primitive streak and head fold form at the beginning of mouse embryo development are *goosecoid* and *Foxa2* (Tam and Behringer, 1997). We applied a *Pf-FoxA* sequence from our previous study (Taguchi et al., 2000) and isolated *Pf-Gsc* sequences from our EST libraries (Tagawa et al., 2014) and asked, using *in situ* hybridization to mRNA, if and when these genes are expressed in developing blastema. We observed no signals in tissue before the blastema formed at 3 dpa. Signals from both *Pf-Gsc* and *Pf-FoxA* then appear in the antero-ventral margin of the blastema in a subset of the tissue expressing *Pf-SoxB1* and *Pf-Klf1/2/4* (Figure 7). Signals for the organizer genes were in the ventral blastema, opposite the dorsal *Pf-Pou3* and *Pf-Msxlx* signals (compare Figures 4B,C and Figures 4F-H with Figures 7A-H). These results indicate that differentiation in the blastema for head regeneration in *P. flava* begins by expression of the same genes as are expressed in pluripotent mouse epiblast cells, as the primitive streak forms and development begins. This result supports the idea that cells establishing the blastema may be pluripotent, like mouse epiblast cells.

## DISCUSSION

Although hemichordates are marine invertebrates, closely related to chordates, including humans, they possess a remarkable regeneration capability, similar to that of planarians. We have shown here that dividing cells in intact animals are mainly those of the gut epithelium, probably because intestinal tissues are continuously being replaced as part of normal homeostasis. The dividing cells do not seem to be stem-like cells that contribute to wound healing or regeneration in hemichordates (Figures 2, 3). We find no evidence to suggest that acorn worms with a primordial deuterostome body plan have a stem-cell-like system resembling that of planaria. Decapitation, followed by initiation of head regrowth appears to activate and mobilize cell division in a population of cells that experience few cell-divisions in intact worms (Figure 3C). These slowly dividing or possibly non-dividing cells are mobilized and contribute as rapidly dividing cells. They are found in the epithelium, blastema and regenerating tissue in decapitated worms (Figures 3C,D). If these cells are a variety of stem-like cells, as seems likely, they do not constitute a prominent portion of regularly dividing cells, although other possibilities for the origin of dividing cells remain to be examined.

The discoveries of mouse and human pluripotent stem cells have engendered much optimism concerning their possible contribution to reparative medicine (Takahashi and Yamanaka, 2006; Takahashi et al., 2007). Very exciting advances establishing the gene programs that determine the pluripotent cell state and the ability to use the key genes as reprogramming factors have raised hopes of making almost any cell become pluripotent. However, to date little success has been achieved in using pluripotent stem cells in regenerative medicine. Studies of modest examples of regeneration in vertebrates, amphibian limb regeneration, zebra fish brain or tail regeneration, etc., find that pluripotent cells are not involved in these processes, and key genes controlling the pluripotent state are not expressed there (Kragl et al., 2009; Onichtchouk, 2012; Tapia et al., 2012). Although the blastema or regeneration blastema, a zone of undifferentiated progenitors, of vertebrates had been thought to contain a homogeneous group of cells and viewed as a single cell-type with multipotency or pluripotency, current studies have demonstrated that the blastema is a population of heterogeneous lineage-restricted progenitor cells (Kragl et al., 2009; Johnson et al., 2020).

In tunicates, invertebrate chordates, the closest relative of vertebrates, whole-body regeneration has been reported in association with asexual reproduction (Ferrario et al., 2020). Although recently, a new model system for solitary ascidians has just emerged and has shown a novel example of whole-body regeneration (Gordon et al., 2019), its molecular mechanism is unclear. In cephalochordates, the earliest diverging invertebrate chordates, however, whole-body regeneration has not been reported, although limited regeneration capabilities have been reported (Ferrario et al., 2020). In echinoderms, another invertebrate deuterostome taxon, whole-body regeneration has been reported in sea stars in relation to axial patterning (Cary et al., 2019), in addition to extensive regeneration reported in all echinoderm classes. However, there are no reports focusing on reprogramming context in whole-body regeneration, except organ regeneration of holothurian echinoderms (Mashanov et al., 2015).

Here we show that hemichordates, kin to chordates, carry functional homologs of key genes in the mammalian reprogramming factor gene network and express these genes during regeneration. Two of the four reprogramming factors, *Oct4* and *Nanog*, are vertebrate-specific genes and are not found even in invertebrate chordates. Since these genes have evolved more rapidly than other genes in mammalian genomes (Holland et al., 2007), as shown by long branch lengths of molecular trees, careful examination is required to discuss orthology or homology between vertebrate and invertebrate counterparts. Furthermore, interspecific conservation among vertebrate homeodomains of the *Nanog* gene is very low, only 28 of 60 amino acid sequences being conserved (Molnar et al., unpublished data). This contrasts to other NKL subclasses of homeobox genes, such as *Msx*, in which 46 of 60 are conserved. In classification of human homeobox genes (Holland et al., 2007), *Nanog* is related to the human *Msx* genes, but there are no *Msxlx* genes found in vertebrates. Invertebrates have both *Msx* and *Msxlx* in their genomes and vertebrate *Nanog* may have been derived from *Msx* in the NKL families just as invertebrate *Msxlx* may have been derived from *Msx*. In the *P. flava* genomes, *Msxlx* is the one most closely related to human *Nanog*. Therefore, hemichordate *Msxlx* might be a functional substitute of vertebrate *Nanog. Pf-Msxlx* expression during regeneration overlaps with *Pf-Pou3* at the dorsal midline of regenerating worms where cell division remains active. This supports *Pf-Msxlx* as a hemichordate homolog of mammalian *Nanog*, although this conclusion should be examined in future functional studies.

On the relationship between hemichordate *Pou3* and vertebrate *Oct4*, our search showed that the *P. flava* genome contains members of classes II, III, IV, and VI of Pou transcription factors, and the class III *Pf-Pou3* is the most closely related sequence to mammalian class V, to which *Oct4* belongs (Supplementary Figures 3, 4). A recent study on evolution of Pou class genes suggests that both class II and class V Pou genes were most likely derived from class III Pou (Gold et al., 2014; Onichtchouk, 2016). A member of class III Pou called Brn4 (Pou3f4) is unable to generate iPSCs when introduced into mouse embryonic fibroblasts. However, Velychko et al. (2020) demonstrated that Brn4 (Pou3f4) modified by swapping domains with those of Oct4 was able to generate iPSCs, although the efficiency was low compared to wild-type Oct4 (Velychko et al., 2020). In the present study, we showed that *Pf-Pou3* could replace the function of intrinsic mouse *Oct4* in ES cells. Therefore, the *Pf-Pou3* gene is very likely a functional homolog, as well as a sequence homolog of mouse *Oct4*. This suggests that the *P. flava* genome may have a pluripotency reprogramming gene program related to that of mammals. It would be interesting to examine in the future whether other invertebrate class III Pou homologs can replace *Oct4*, as in the case of *Pf-Pou3*.

The most likely interpretation of our results is that hemichordates possess a signaling system that activates a pluripotent reprogramming-like gene network and utilizes this system as part of its wound healing response. This may account for the very vigorous regenerative capabilities of hemichordates. If this is the case, it supports the very optimistic projections that knowing how *P. flava* makes and controls pluripotent cells could be a key to understanding in regenerative biology and improving regenerative medicine. Nevertheless, why do vertebrates other than mammals not use their well-developed pluripotency reprogramming capabilities for regeneration? One interpretation is that the hemichordate situation is probably ancestral in deuterostomes, and that the vertebrate evolutionary line, for currently unfathomable reasons, lost the capability to activate this program in wound responses. The alternative is that hemichordates independently developed signaling pathways to activate pluripotent reprogramming during wound healing responses and to use pluripotent cells to regenerate effectively. Since our results indicate that a great deal of functional homology of reprogramming factors has been conserved during the considerable evolutionary time between hemichordates and mice, either of the above scenarios suggest that the signaling system now present in hemichordates could inform approaches to activate reprogramming and regeneration in human injuries. Further expression analyses of these genes during normal head development and comparisons of expression patterns between normal and regenerating heads may disclose clearer molecular mechanisms underlying potential functions of these genes in evolution of animal regeneration.

## Supplementary Material

Supplemental document

Supplementary figure S1

Supplementary figure S2

Supplementary figure S3

Supplementary figure S4

Supplementary figure S5

Supplementary figure S6

Supplementary tables

## Data Availability

The datasets presented in this study can be found in online repositories. The names of the repository/repositories and accession number(s) can be found in the article/ Supplementary Material.
