# Supplementary material for: Ancestral Stem Cell Reprogramming Genes Active in Hemichordate Regeneration
Source: Front Ecol Evol. Author manuscript; Available in PMC 2023 Mar 31. (PMC10065570; doi:10.3389/fevo.2022.769433)
Supplement: Supplemental document [file NIHMS1836806-supplement-Supplemental_document.docx]

Supplemental Document

**Identification and characterization of *Ptychodera flava* genes used in this study**

Molecular phylogenetic analysis was performed to identify genes encoding the gene record or cDNA clones we used. First, the correspondence between cDNA clones and gene models for the genome sequence was confirmed with BlastN searches (Camacho et al 2008) followed by sequence alignment by Mafft (ver 7.0, Katoh and Standley, 2013). Those correspondences are as follows.

Gene Name, cDNA clone, Gene model

**Pf-SoxB1**, BAO23502.1, pfl_40v0_9_20150316_1g5033.t1

**Pf-Klf1/2/4**, Pfh4d48l03, pfl_40v0_9_20150316_1g6400.t1

**Pf-Pou3**, Pfemg35m19, pfl_40v0_9_20150316_1g4969.t1

**Pf-Msxlx**, Pfemg29g03, pfl_40v0_9_20150316_1g13652.t1

**Pf-FoxA**, BAA82786.1, pfl_40v0_9_20150316_1g1338.t1

**Pf-Gsc**, LC622255, pfl_40v0_9_20150316_1g3238.t1

Molecular phylogenetic analyses of gene families into which the above genes are classified have already been reported: Sox family (Bowles et al 2000), KLF family (Shao et al., 2017, Li et al., 2011), POU family (Zhang et al., 2013), Msxlx (Butts et al., 2010), Fox family (Mazet et al., 2003, Phochanukul and Russell 2010), and Goosecoid (Neidert et al., 2000). Therefore, we collected amino acid sequences based on those reports and used them as initial query sequences to identify genes. We omitted phylogenetic analyses of Pf-SoxB1, Pf-FoxA and Pf-Gsc since those data are available in other reports (Taguchi et al., 2002 and Zhong et al., 2011 for SoxB1; Taguchi et al., 2000 and Simakov et al. 2015 for FoxA; Su et al., 2019 for Gsc).

Target organisms for gene collection were human beings (*Homo sapiens*), mice (*Mus musculus*), ascidians (*Ciona intestinalis*), amphioxus (*Branchiostoma floridae*), two species of acorn worm (*Ptychodera flava*, *Saccoglossus kowalevskii*), sea urchins (*Strongylocentrotus purpuratus*), and a sea anemone (*Nematostella vectensis*). Gene model sets used were as follows.

**Species, Databases, Proteome versions, References**

***H. sapiens***: Ensembl, Homo_sapiens.GRCh38.pep.all.fa (Yates., et al 2018)

***M. musculus***: Ensembl, Mus_musculus.GRCm38.pep.all.fa (Yates., et al 2018)

***C. intestinalis***: Ghost Database (Kyoto), KH.KHGene.2013.Longest.protein.mod.fasta (Satou et al., 2005)

***B. floridae:*** JGI genome database, proteins.Brafl1.fasta (Putnam et al., 2008)

***P. flava***: OIST Marine genomics database, pfl_public_ver1.0.prot (Shimakov et al., 2015)

***S. kowalevskii***: JGI genome database, SkowalevskiiJGIv3.0.longestTrs.pep.fa (Shimakov et al., 2015).

***S. purpuratus***: Echinobase, SPU_peptide.fasta (Kudtarkar and Cameron 2009)

***N. vectensis***: JGI genome database, proteins.Nemve1FilteredModels1.fasta (Putnam et al., 2007)

Genes with high sequence similarity (1e-^20^ < by BlastP) were collected from these genomes. Multiple alignments were performed using Mafft, followed by AliView (Larsson 2014) to remove gaps. Molecular phylogenetic analysis was performed using the neighbor-joining method (ClustalW2 with the tree option) (Larkin et al., 2007). However, when all these genes are used to construct a phylogenetic tree, the consensus sequence of a multiple alignment becomes extremely short due to deletion of gap regions. To avoid this problem, we developed a two-step method for phylogenetic tree construction.

As a first step, we created a phylogenetic tree using all genes with a short consensus sequence. Based on the first phylogenetic tree, we removed genes that were not included in the same clade as reported orthologs in other species. Some of the above gene models provide alleles and splicing variants as different gene models. In such cases, we kept the longest one and deleted the rest.

Based on the genes remaining after these operations, we performed a second step of phylogenetic tree construction. We again performed multiple alignments using Mafft, and then used AliView to remove gaps. The results of the second alignment are shown in Supplemental Files S1 to S3. Gene model IDs and gene names used in the alignments are listed in Supplemental Tables S1 to S3. According to the usual method for preparing the phylogeny, all gap-regions in the multiple-alignment were removed. Positions of the remaining amino acid sequences are listed in Supplementary Table S4. Positions in Supplementary Table S4 are not the positions after alignment, but positions of the original amino acid sequences of *P. flava,* as a representative. Then we performed molecular phylogenetic analysis using the neighbor-joining method with the tree option of ClustalW2. Bootstrap values for each node of the phylogenetic tree were calculated using the -BOOTSTRAP option of ClustalW2 as follows.

clustalw2 -infile="<alignment-file>" -BOOTSTRAP=1000 ^**^

We also performed phylogenetic tree searches using the maximum likelihood method on the same data aligned with RAxML (Ver 8.2.9, Stamatakis (2014)). Options used in the calculations for each alignment data are as follows.

raxmlHPC-PTHREADS -f a -T 4 -x 12345 -p 12345 -# 1000 -m PROTGAMMAMTREVF -s <alighment-file>-n <alinment-file>.bs1000 ^**^

Then,

raxmlHPC-PTHREADS -f b -m PROTGAMMAMTREVF -t RAxML_bootstrap.<alignment-file>.bs1000 -z RAxML_bootstrap.<alignment-file>.bs1000 -n BOOTSTRAP ^**^

** Where it says "<alignment-file>", it corresponds to each file name of the multiple alignment edited in AliView.

Results of molecular phylogenetic analysis by the neighbor joining and maximum likelihood methods are shown in Supplemental Figures S1 to S6. Bootstrap values >80% are shown in green at nodes for all genes. Asterisks in each panels show *P. flava* sequences used in this study.

**Results of phylogenetic trees**

**Supplementary Figures S1 and S2.**

Molecular phylogenetic analysis confirmed that one gene model of *P. flava* was in the same clade as those of humans, mice and sea urchin KLF1/2/4, although the bootstrap value was not sufficient to support orthology. However, we found a Klf1/2/4-specific motif in the N-terminal region of the *P. flava* sequence. Therefore, this gene model was designated as Pf-Klf1/2/4.

**Supplementary Figures S3 and S4.**

The sequence we used as Pf-Pou3 was in the same clade as human POU3F1/F2/F3/F4, shown in the molecular phylogenetic analysis. In addition, we searched for linker sequences between the two DNA binding domains, the POU domain and the homeodomain, of Pf-Pou3 and found class III POU-specific linker sequences “STTGSPTSIDKIAAQGR” (Cheng et al., 2014). These sequences are broadly conserved among bilaterians. Therefore, we decided to use this gene model as Pf-Pou3.

**Supplementary Figures S5 and S6.**

Pf-Msxlx was easily identified due to conservation of gene order (Simakov et al., 2015). Additionally, we also performed molecular phylogenetic analysis using Msxlx from sea urchins, amphioxus, red flour beetles, and *Drosophila*, to confirm the gene identity. We found that Pf-Msxlx is in the same clade as the Msxlx of *S. purpuratus*. Finally, we designated Pf-Msxlx as the Msxlx of *P. flava*.

**References**

Bowles J., Schepers G., Koopman P. Phylogeny of the SOX family of developmental transcription factors based on sequence and structural indicators. Dev. Biol. 2000, 227, 239-255.

Butts T., Holland P.W.H., Ferrier D.E.K. Ancient homeobox gene loss and the evolution of chordate brain and pharynx development: deductions from amphioxus gene expression. Proc. R. Soc. 2010, B 277, 3381-3389.

Camacho C., Coulouris G., Avagyan V., Ma N., Papadopoulos J., Bealer K., & Madden T.L. BLAST+: architecture and applications. BMC Bioinformatics 2008, 10:421. doi: 10.1186/1471-2105-10-421.

Cheng, C.C.; Ko, A.; Chaieb, L.; Koyama, T.; Sarwa1, P.; Mirth, C.K.; Smith, W.A.; Suzuki, Y. The POU factor ventral veins lacking/Drifter directs the timing of metamorphosis through ecdysteroid and juvenile hormone signaling. PLoS Genet. 2014, 10(6):e1004425. doi: 10.1371/journal.pgen.1004425.

Katoh K., Standley D.M. MAFFT multiple sequence alignment software version 7: improvements in performance and usability. Mol. Biol. Evol. 2013, 30, 772-780.

Kudtarkar P., Cameron R.A. (2017). Echinobase: an expanding resource for echinoderm genomic information. Database: bax074. doi: 10.1093/database/bax074.

Larkin M.A., Blackshields G., Brown N.P., Chenna R., McGettigan P.A., McWilliam H., Valentin F., Wallace I.M., Lopez R., Thompson J.D., Gibson T.J., Higgins D.G. Clustal W and Clustal X version 2.0. Bioinformatics. 2007, 23(21), 2947-2948. doi: 10.1093/bioinformatics/btm404.

Larsson A. AliView: a fast and lightweight alignment viewer and editor for large datasets. Bioinformatics. 2014, 30(22), 3276-3278. doi: 10.1093/bioinformatics/btu531.

Li I. Chan C., Lu Y., Wu Y., Chen Y., Li G., Lin C., Hwang S.L. Zebrafish Kruppel-like factor 4a represses intestinal cell proliferation and promotes differentiation of intestinal cell lineages. PLoS One. 2011, 6(6):e20974. doi: 10.1371/journal.pone.0020974.

Mazet F., Yu J, Liberles D.A., Holland L.Z., Shimeld S.M. Phylogenetic relationships of the Fox (Forkhead) gene family in the Bilateria. Gene. 2003, 316, 79-89.

Neidert A.H., Panopoulou G., Langeland J.A. Amphioxus goosecoid and the evolution of the head organizer and prechordal plate. Evol. Dev. 2000, 2(6), 303-310.

Phochanukul N., Russell S. No backbone but lots of Sox: Invertebrate Sox genes. Int. J. Biochem. Cell Biol. 2010, 42, 453-464.

Putnam, N., Butts, T., Ferrier, D.E.K., Furlong, R.F., Hellsten, U., Kawashima, T., Robinson-Rechavi, M., Shoguchi, E., Terry, A., Yu, J.K., Benito-Gutiérrez, E., Dubchak, I., Garcia-Fernàndez, J., Gibson-Brown, J.J., Grigoriev, I.V., Horton, A.C., de Jong PJ, Jurka J, Kapitonov VV, Kohara Y, Kuroki Y, Lindquist E, Lucas S, Osoegawa K, Pennacchio KA, Salamov AA, Satou Y, Sauka-Spengler T, Schmutz J, Shin-I T, Toyoda A, Bronner-Fraser M, Fujiyama A, Holland LZ, Holland PWH, Satoh N, Rokhsar DS. The amphioxus genome and the evolution of the chordate karyotype. Nature. 2008, 453, 1064-1071.

Putnam N.H., Srivastava M., Hellsten U., Dirks B., Chapman J., Salamov A., Terry A., Shapiro H., Lindquist E., Kapitonov V.V., Jurka J., Genikhovich G., Grigoriev I.V., Lucas S.M., Steele R.E., Finnerty J.R., Technau U., Martindale M.Q., Rokhsar D.S. (2007). Sea Anemone Genome Reveals Ancestral Eumetazoan Gene Repertoire and Genomic Organization. Science. 2014, 317, 5834, 86-94.

Satou Y. Kawashima T., Shoguchi E., Nakayama A., Satoh N. An integrated database of the ascidian, *Ciona intestinalis*: towards functional genomics. Zoolog. Sci. 2005, 22(8), 837-843.

Shao M., Ge G., Liu W., Xiao J., Xia H., Fan Y., Zhao F., He B., Chen C. Characterization and phylogenetic analysis of Kruppel-like transcription factor (KLF) gene family in tree shrews (*Tupaia belangeri chinensis*). Oncotarget. 2017, 8(10), 16325-16339.

Simakov, O., Kawashima T., Marlétaz F., Jenkins J., Koyanagi R., Mitros T., Hisata K., Bredeson, J., Shoguchi, E., Gyoja, F., Yue, J.X., Chen, Y.C., Freeman RM., Sasaki A., Hikosaka-Katayama, T., Sato, A., Fujie, M., Baughman, K.W., Levine, J., Gonzalez, P., Cameron, C., Fritzenwanker, J.H., Pani, A.M., Goto, H., Kanda, M., Arakaki, N., Yamasaki, S., Qu, J., Cree, A., Ding, Y., Ding, H.H., Dugan, S., Holder, M., Jhangiani, S.N., Kovar, C.L., Lee, SL., Lewis, L.R., Morton, D., Nazareth, L.V., Okwuonu, G., Santibanez, J., Chen, R., Richards, S., Musky, D.M., Gillis, A., Peshkin L., Wu, M., Humphreys, T., Su, YH., Putnam N.H., Schmutz, J., Fujiyama, A., Yu, JK., Tagawa, K., Worley KC., Gibbs, R.A., Kirschner M.W., Lowe, C.J., Satoh, N., Rokhsar, D.S., Gerhart, J. Hemichordate genomes and deuterostome origins. Nature. 2015, 527, 459-465.

Stamatakis, A. RAxML Version 8: a tool for phylogenetic analysis and post-analysis of large phylogenies. Bioinformatics. 2014, 30(9): 1312-1313.

Su, Y.H.; Chen, Y.C.; Ting, H.C.; Fan, T.P.; Lin, C.Y.; Wang, K.T.; and Yu, J.K. BMP controls dorsoventral and neural patterning in indirect-developing hemichordates providing insight into a possible origin of chordates. Proc Natl Acad Sci U S A. 2019, 116(26):12925-12932. doi: 10.1073/pnas.1901919116.

Taguchi, S., Tagawa, K., Humphreys, T., Nishino, A., Satoh, N., Harada, Y. Characterization of a hemichordate fork head/HNF-3 gene expression. Dev. Genes Evol. 2000, 210 (1), 11-17.

Taguchi, S.; Tagawa, K.; Humphreys, T.; Satoh, N. Group B Sox genes that contribute to specifications of the vertebrate brain are expressed in the apical organ and ciliary bands of hemichordate larvae. Zoolog. Sci. 2002, 19, 57-66.

Yates A.D., Achuthan P., Akanni W., Allen J., Allen J., Alvarez-Jarreta J., Amode M.R., Armean I.M., Azov A.G., Bennett R., Bhai J., Billis K., Boddu S., Marugán J.C., Cummins C., Davidson C., Dodiya K., Fatima R., Gall A., Giro’n C.G., Gil L., Grego T., Haggerty L., Haskell E., Hourlier T., Izuogu O.G., Janacek S.H., Juettemann T., Kay M., Lavidas I., Le T., Lemos D., Martinez J.G., Maurel T., McDowall M., McMahon A., Mohanan S., Moore B., Nuhn M., Oheh D.N., Parker A., Parton A., Patricio M., Sakthivel M.P., Salam A.I.A., Schmitt B.M., Schuilenburg H., Sheppard D., Sycheva M., Szuba M., Taylor K., Thormann A., Threadgold G., Vullo A., Walts B., Winterbottom A., Zadissa A., Chakiachvili M., Flint B., Frankish A., Hunt S.E., IIsley G., Kostadima M., Langridge N., Loveland J.E., Martin F.J., Morales J., Mudge J.M., Muffato M., Perry E., Ruffier M., Trevanion S.J., Cunningham F., Howe K.L., Zerbino D.R., Flicek P. Ensembl 2020. Nuc. Acid. Res. 2020, 48:D1, D682-D688.

Zang X., Ma Y., Liu X., Zhou Q., Wang X. Evolutionary and functional analysis of the key pluriptency factor oct4 and its family proteins. J. Genet Genomics. 2013, 40, 399-412.

Zhong, L.; Wang, D.; Xiaoni Gan, X.; Yang,T.; He, S. Parallel expansions of Sox transcription factor group B predating the diversifications of the arthropods and jawed vertebrates. PLoS One. 2011, 6(1):e16570. doi: 10.1371/journal.pone.0016570.
