# Supplementary figures and images for: Ancestral Stem Cell Reprogramming Genes Active in Hemichordate Regeneration
Source: Front Ecol Evol. Author manuscript; Available in PMC 2023 Mar 31. (PMC10065570; doi:10.3389/fevo.2022.769433)

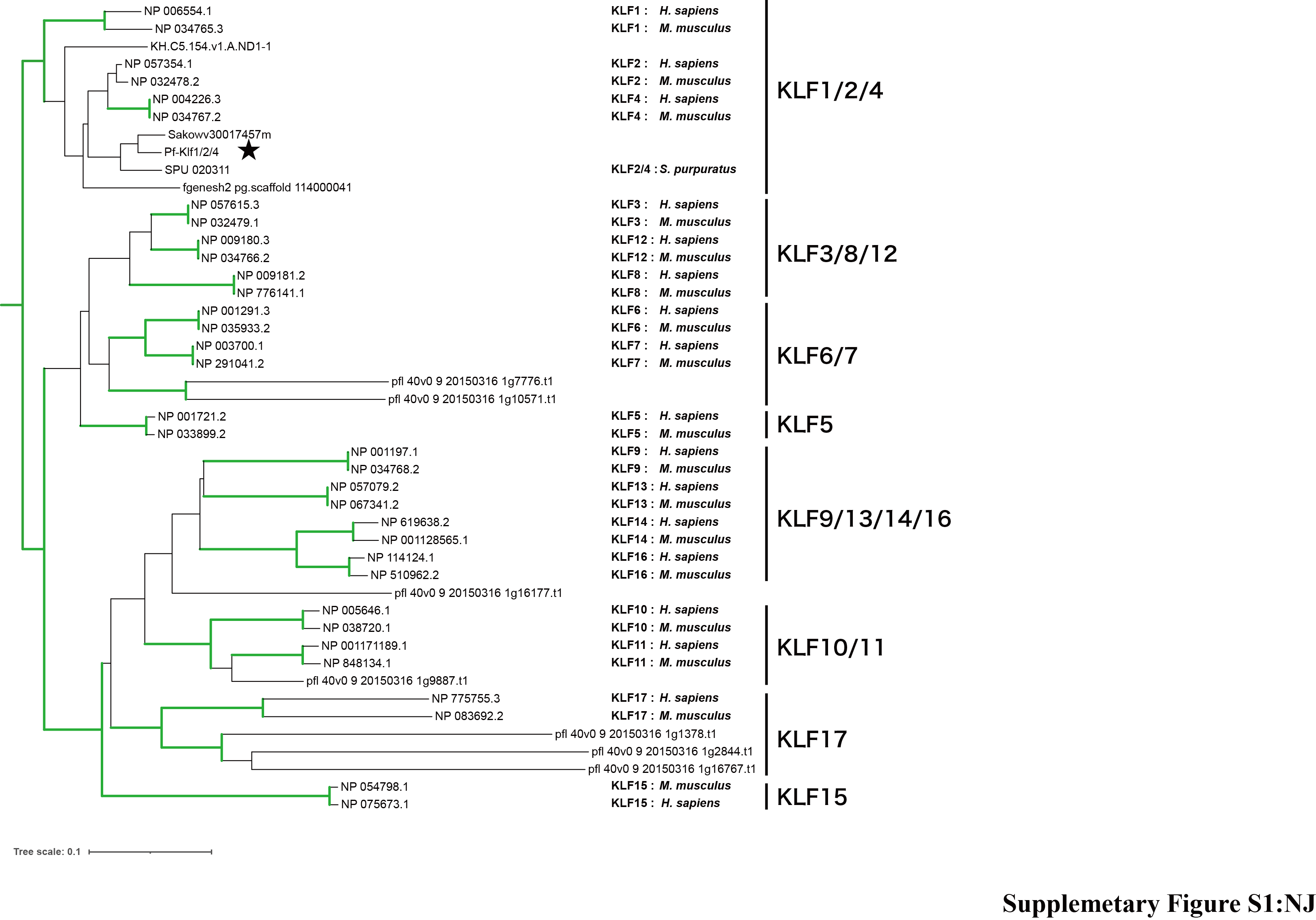

Supplement: Supplementary figure S1 [file NIHMS1836806-supplement-Supplementary_figure_S1.png]

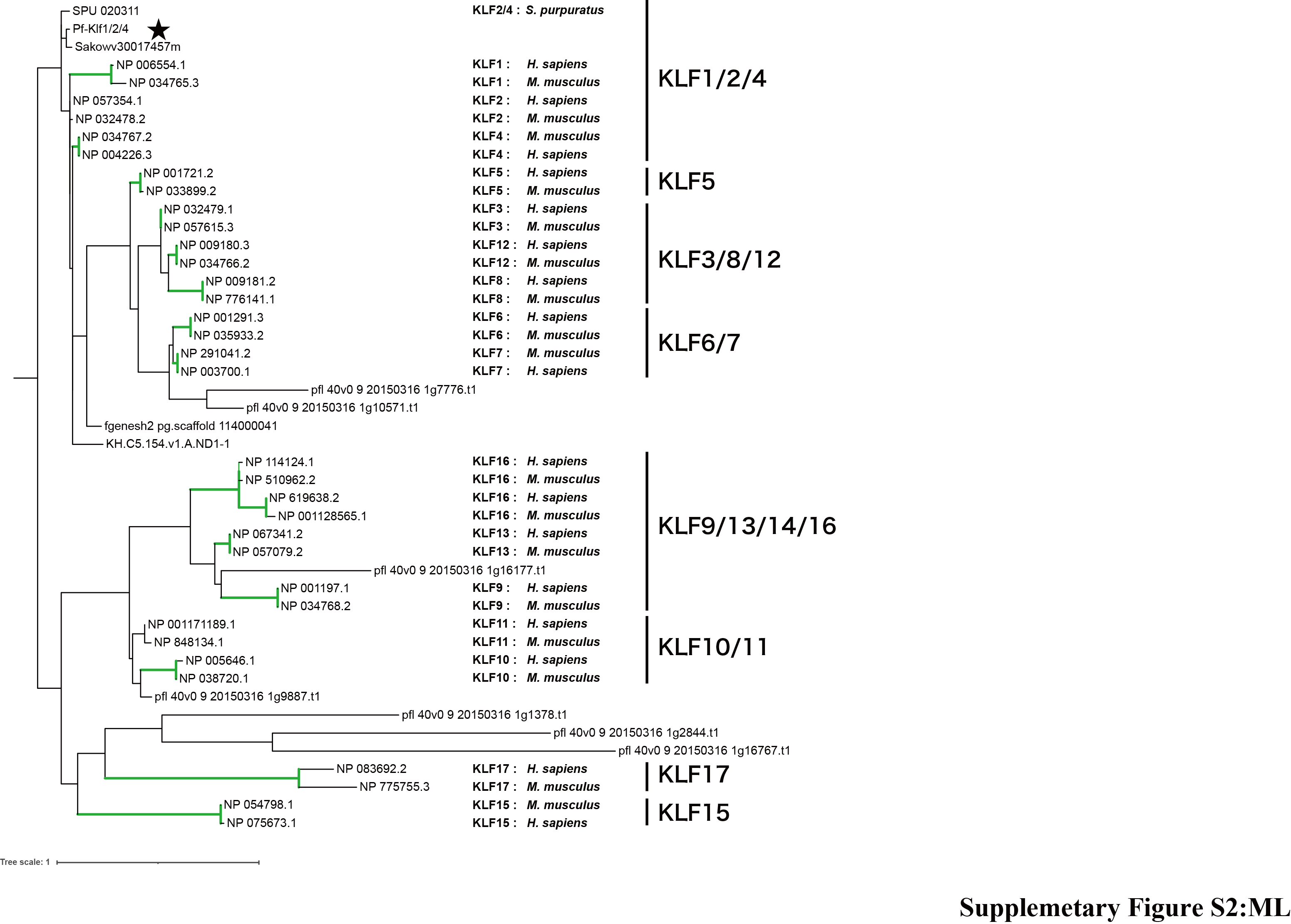

Supplement: Supplementary figure S2 [file NIHMS1836806-supplement-Supplementary_figure_S2.png]

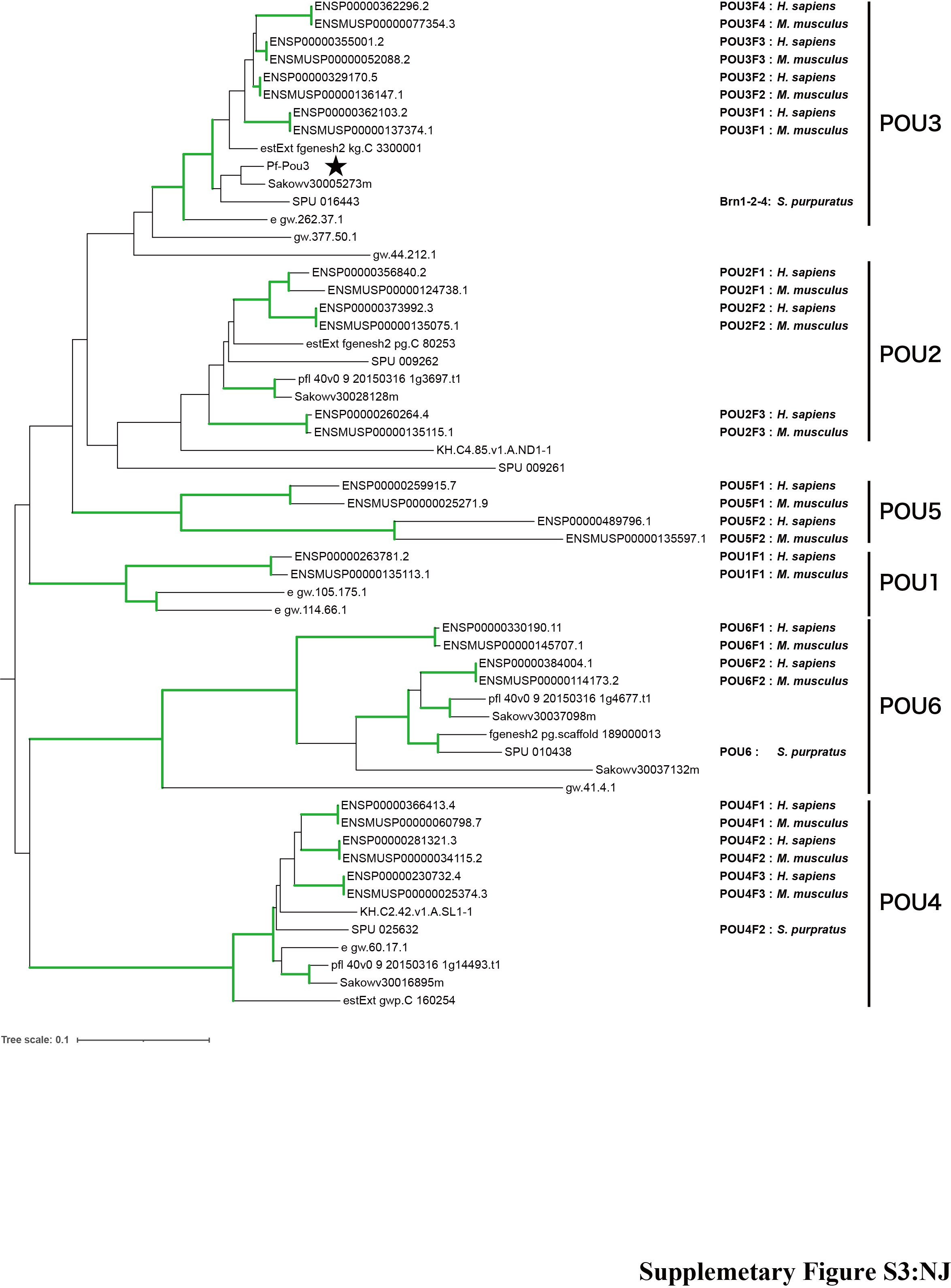

Supplement: Supplementary figure S3 [file NIHMS1836806-supplement-Supplementary_figure_S3.png]

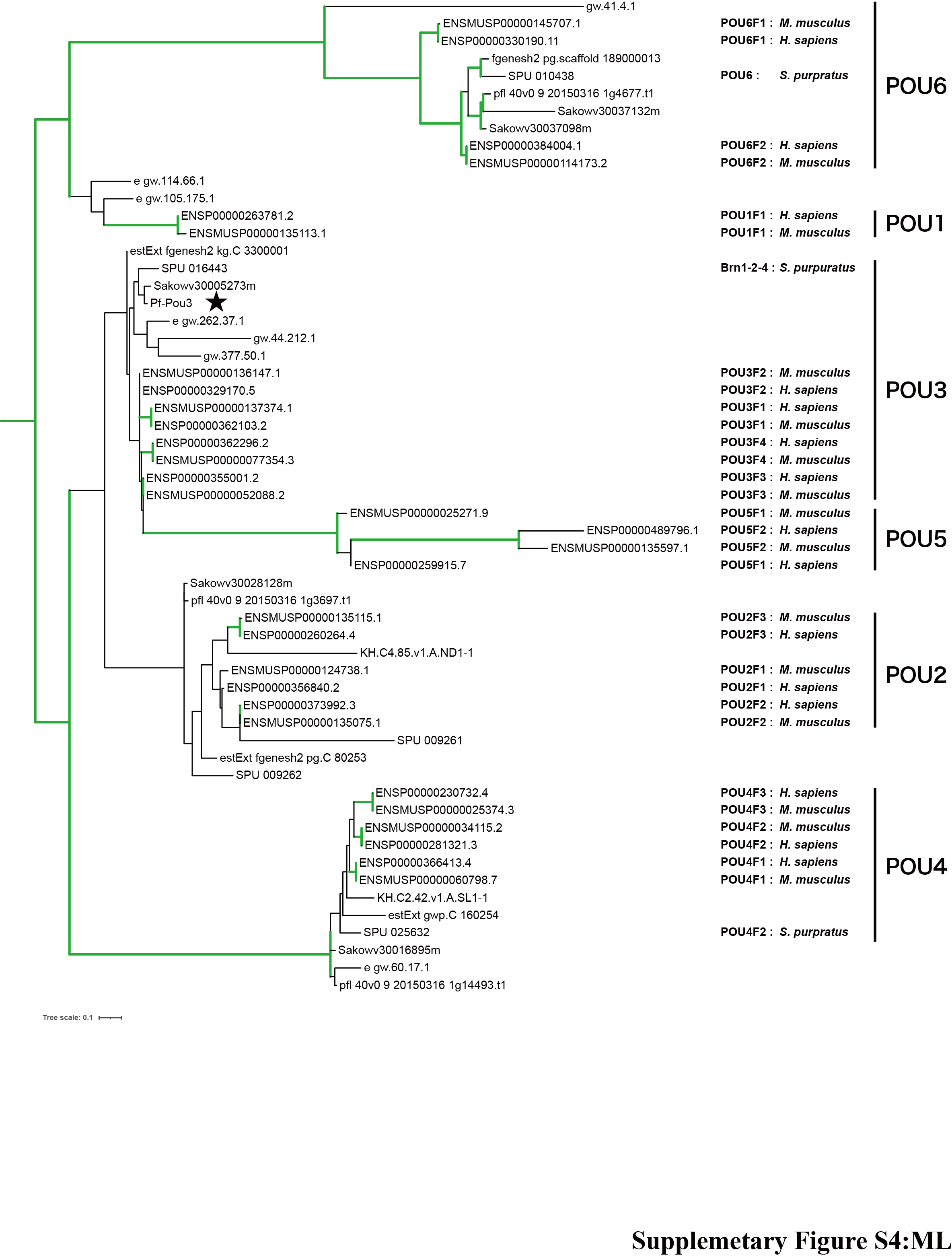

Supplement: Supplementary figure S4 [file NIHMS1836806-supplement-Supplementary_figure_S4.png]

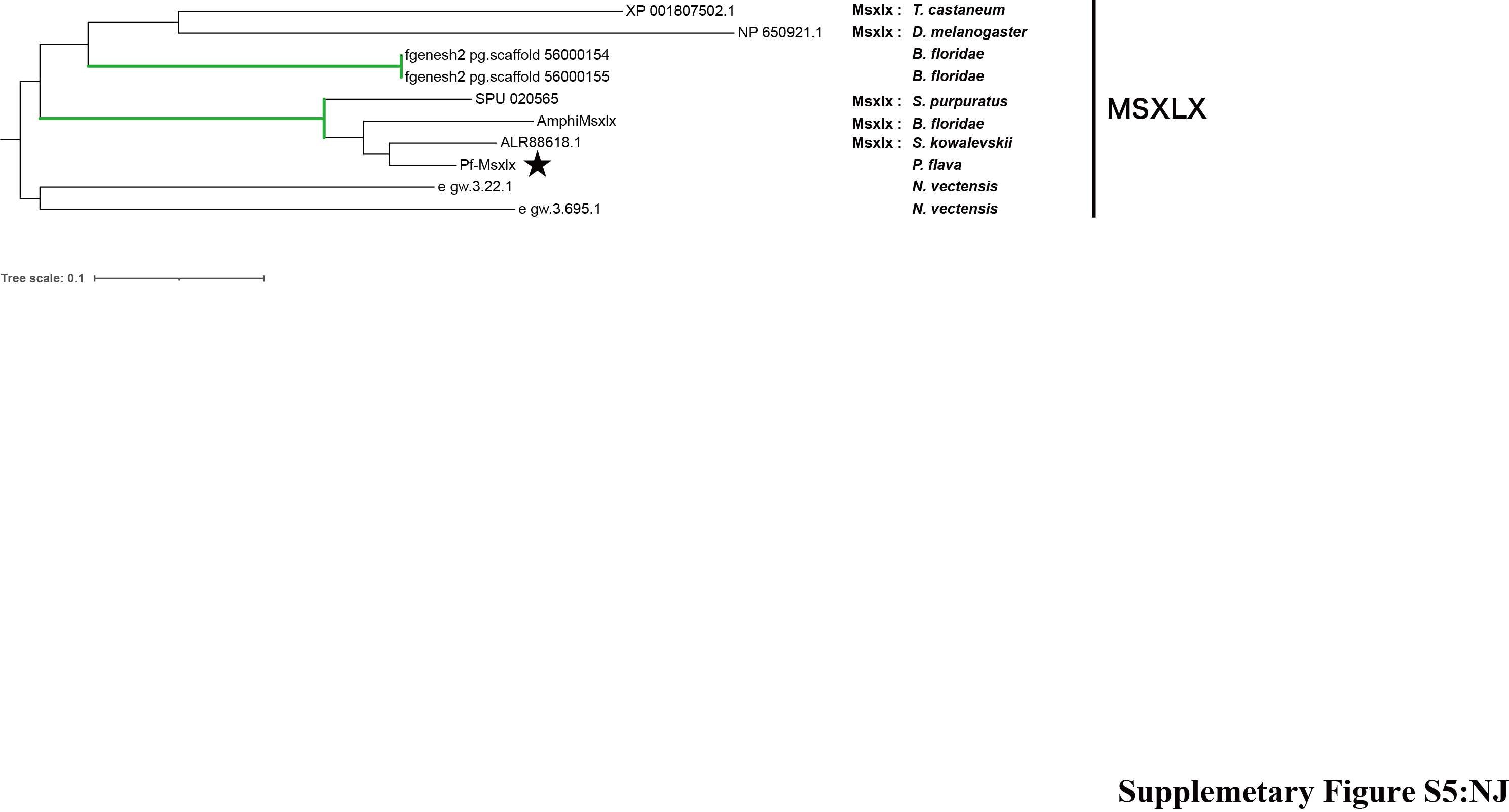

Supplement: Supplementary figure S5 [file NIHMS1836806-supplement-Supplementary_figure_S5.png]

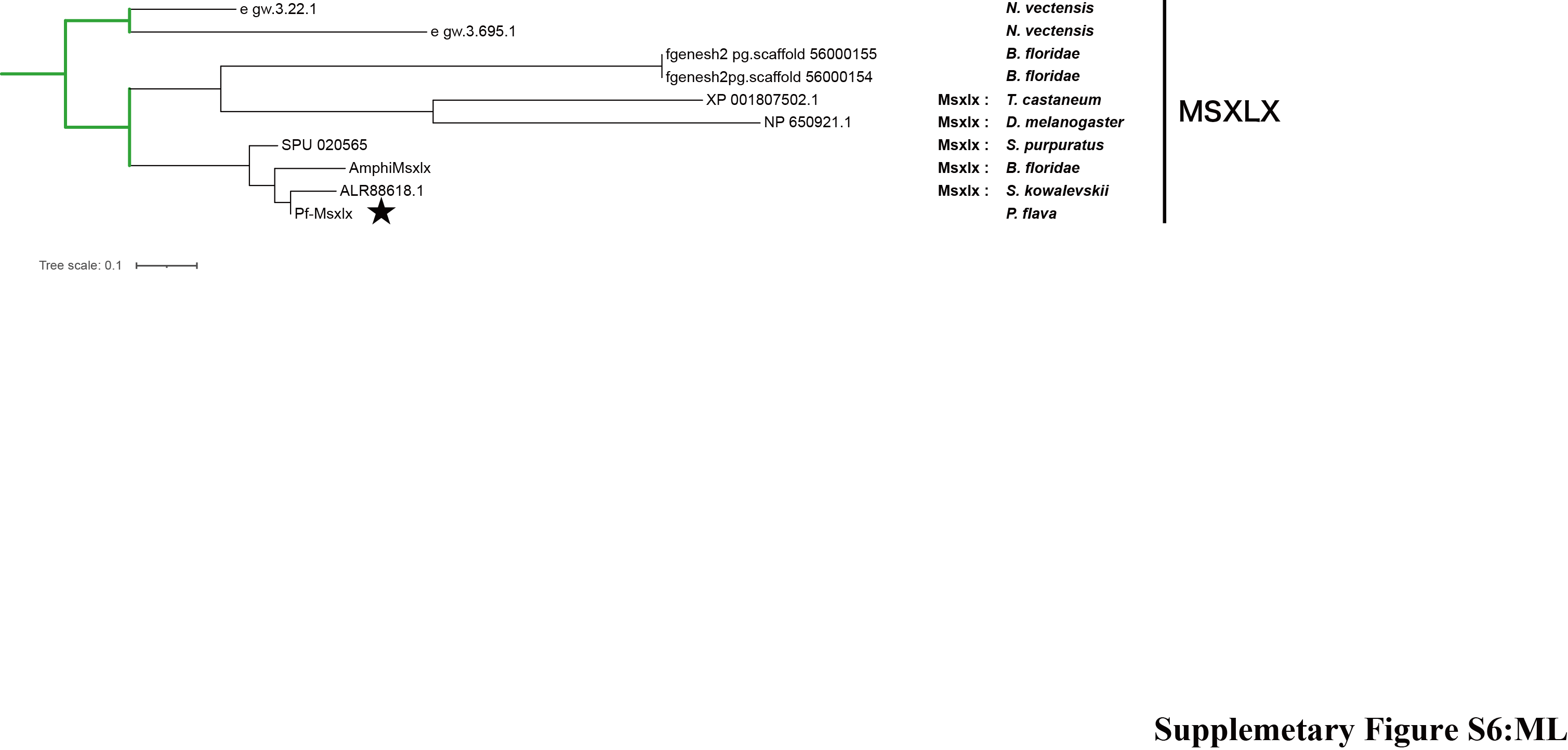

Supplement: Supplementary figure S6 [file NIHMS1836806-supplement-Supplementary_figure_S6.png]
